# The Evolutionary Success of the Marine Bacterium SAR11 Analyzed through a Metagenomic Perspective

**DOI:** 10.1128/mSystems.00605-20

**Published:** 2020-10-06

**Authors:** Mario López-Pérez, Jose M. Haro-Moreno, Felipe Hernandes Coutinho, Manuel Martinez-Garcia, Francisco Rodriguez-Valera

**Affiliations:** a Evolutionary Genomics Group, División de Microbiología, Universidad Miguel Hernández, San Juan, Alicante, Spain; b Department of Physiology, Genetics, and Microbiology, University of Alicante, Alicante, Spain; c Research Center for Molecular Mechanisms of Aging and Age-Related Diseases, Moscow Institute of Physics and Technology, Dolgoprudny, Russia; University of Hawaii at Manoa

**Keywords:** SAR11, microdiversity, evolution, metagenomics, homologous recombination, intrapopulation diversity, evolutionary dynamics

## Abstract

The SAR11 clade of *Alphaproteobacteria* is the most abundant group of planktonic cells in the near-surface epipelagic waters of the ocean, but the mechanisms underlying its exceptional success have not been fully elucidated. Here, we applied a metagenomic approach to explore microdiversity patterns by measuring the accumulation of synonymous and nonsynonymous mutations as well as homologous recombination in populations of SAR11 from different aquatic habitats (marine epipelagic, bathypelagic, and surface freshwater). The patterns of mutation accumulation and recombination were compared to those of other groups of representative marine microbes with multiple ecological strategies that share the same marine habitat, namely, *Cyanobacteria* (*Prochlorococcus* and *Synechococcus*), *Archaea* (“*Candidatus* Nitrosopelagicus” and Marine Group II *Thalassoarchaea*), and some heterotrophic marine bacteria (*Alteromonas* and *Erythrobacter*). SAR11 populations showed widespread recombination among distantly related members, preventing divergence leading to a genetically stable population. Moreover, their high intrapopulation sequence diversity with an enrichment in synonymous replacements supports the idea of a very ancient divergence and the coexistence of multiple different clones. However, other microbes analyzed seem to follow different evolutionary dynamics where processes of diversification driven by geographic and ecological instability produce a higher number of nonsynonymous replacements and lower intrapopulation sequence diversity. Together, these data shed light on some of the evolutionary and ecological processes that lead to the large genomic diversity in SAR11. Furthermore, this approach can be applied to other similar microbes that are difficult to culture in the laboratory, but abundant in nature, to investigate the underlying dynamics of their genomic evolution.

**IMPORTANCE** As the most abundant bacteria in oceans, the *Pelagibacterales* order (here SAR11) plays an important role in the global carbon cycle, but the study of the evolutionary forces driving its evolution has lagged considerably due to the inherent difficulty of obtaining pure cultures. Multiple evolutionary models have been proposed to explain the diversification of distinct lineages within a population; however, the identification of many of these patterns in natural populations remains mostly enigmatic. We have used a metagenomic approach to explore microdiversity patterns in their natural habitats. Comparison with a collection of bacterial and archaeal groups from the same environments shows that SAR11 populations have a different evolutionary regime, where multiple genotypes coexist within the same population and remain stable over time. Widespread homologous recombination could be one of the main driving factors of this homogenization.

## Abstract (precis)

As the most abundant bacteria in oceans, the *Pelagibacterales* order (here SAR11) plays an important role in the global carbon cycle, but the study of the evolutionary forces driving its evolution has lagged considerably due to the inherent difficulty of obtaining pure cultures. Multiple evolutionary models have been proposed to explain the diversification of distinct lineages within a population; however, the identification of many of these patterns in natural populations remains mostly enigmatic. We have used a metagenomic approach to explore microdiversity patterns in their natural habitats. Comparison with a collection of bacterial and archaeal groups from the same environments shows that SAR11 populations have a different evolutionary regime, where multiple genotypes coexist within the same population and remain stable over time. Widespread homologous recombination could be one of the main driving factors of this homogenization.

## INTRODUCTION

The open ocean is one of the largest and most biologically productive microbial habitats in the biosphere, and marine microbial communities have an essential role in global biogeochemical cycling (1). The development of culture-free approaches such as metagenomics has significantly advanced our knowledge of the geographical distribution (2, 3), seasonal dynamics (4–6), and vertical distribution throughout the water column (7, 8) of these communities. In addition, we are now starting to understand the real complexity of microbial populations within natural environments. Thus, recent advances in single-cell sequencing have offered a new view of the microbial genomic diversity in unprecedented detail (9). The reconstruction of genomes from uncultivated microbes using metagenomes, referred to as metagenome-assembled genomes (MAGs) or single-amplified genomes (SAGs), obtained by sequencing individual cells has improved our understanding of the microbial diversity, evolution, and ecology of these microbes (10, 11). These complementary approaches (metagenomics and single-cell sequencing) have shown that, in nature, microbes live in populations made up of complex consortia of different clonal lineages (12–14). The vast diversity of the genetic pool found within single populations has been one major discovery brought up by such technologies (15, 16).

Mapping metagenomic reads against reference genomes has been applied to capture the heterogeneity at the genomic level within natural populations (17, 18). These reads are derived from strains closely related to the strain of the reference genome that are concurrent within a sample. For most marine microbes, the minimum alignment identity threshold of the vast majority of reads assigned to the core genome is located at above 95% identity, delimiting what has been termed “sequence-discrete” populations (19–21). However, these threshold values, which have also been used for the delimitation of species (22), should be taken with caution (23). For example, an exception to this rule is the populations of the SAR11 clade. For them, the threshold goes down to ca. 92% (24) or even lower (ca. 87%) based on a more recent study (25). These variations can be analyzed at single-nucleotide resolution (i.e., microdiversity) (26). In addition, intrapopulation microdiversity has also been proposed as a measure of evolutionary success. High diversity corresponds to populations with high temporal persistence and therefore greater adaptive success in their environment (17, 27). In contrast, less diversity is associated with populations that have undergone a recent clonal sweep (18, 27).

Despite being the most abundant and successful marine microorganisms in the surface ocean (28), the study of SAR11 population structures and dynamics has lagged considerably due to the difficulty of culturing and isolating these organisms and the paradoxical difficulty of obtaining MAGs for this group, as demonstrated by several marine metagenomic studies around the world (8, 29, 30). Single-cell genomics overcame some of these limitations of metagenomic assembly and promoted the increase in the number of individual genomes sequenced of the SAR11 clade, thus disentangling part of the elusive genetic diversity among members of this taxon (24, 31–36). Although the delimitation of populations within this clade is a controversial issue, in a recent study, an improved phylogenomic classification (enriched by single-cell genomes) based on whole-genome comparisons, together with a fine ecogenomic characterization of SAR11 at a global scale, allowed discerning novel operational taxonomic units, which were called genomospecies (33). Genomes within these genomospecies showed remarkable agreement between their phylogenomic classification and patterns of metagenomic distribution across different metagenomes, displaying a minimum pairwise average nucleotide identity (ANI) value within genomospecies of ca. 80% (33). In that study, these genomospecies were used to define SAR11 populations (i.e., cells belonging to the same genomospecies). A total of 20 genomospecies were differentiated within nine phylogenomic subclades. Subclade Ia.3 was particularly well represented, with the largest number of genomes (47, including 6 pure cultures), and due to the high read recruitment to these genomes in the available metagenomic data sets, they could be split into 6 well-defined genomospecies with different spatiotemporal abundance patterns (33).

Recently, a complementary metagenomic approach using single amino acid variants has been used to investigate evolutionary processes that maintain genetic diversity within subclade Ia.3/V through a single isolate genome, HIMB83 (25). Results showed patterns of amino acid diversity driven by large-scale ocean circulation. Other studies using genome comparison and phylogenomic approaches have estimated that while marine SAR11 isolates showed recombination rates that were among the highest reported in bacteria (37, 38), freshwater SAR11 isolates had much lower values (34). Here, we have used a metagenomic approach to investigate the patterns of sequence microdiversity and try to understand the evolutionary forces driving the evolution within and among these six subclade Ia.3 genomospecies (here, the term “population” is applied to groups of individual cells belonging to the same genomospecies and sharing the same habitat, i.e., potentially exchanging DNA). In addition, in order to compare the pictures provided by these epipelagic dwellers, we included genomospecies Ib.1/III and Ib.2/I, which belong to subclade Ib (also epipelagic), the deep-ocean bathytype (subclade Ic) (32), and the freshwater lineage LD12 (subclade IIIb) (39). We have also analyzed the evolutionary dynamics of groups of microbes that cohabit the water column with SAR11, such as *Cyanobacteria* (*Prochlorococcus* and *Synechococcus*), *Archaea* (“*Candidatus* Nitrosopelagicus” and Marine Group II *Thalassoarchaea*), and some heterotrophic marine bacteria (*Alteromonas* and *Erythrobacter*). Our results suggest a different process of bacterial diversification in SAR11 populations in comparison to the other microbes analyzed, which is in agreement with a metastable model (40), that is, one in which frequent recombination keeps the population relatively stable while maintaining high intrapopulation diversity of mostly synonymous replacements.

## RESULTS

### Microdiversity within the Ia.3 subclade.

First, we studied microevolution among six genomospecies within the Ia.3 subclade using metagenomic read mapping to measure the ratio of nonsynonymous to synonymous polymorphisms (*pN/pS* ratio) (26). Figure S1 shows the phylogenomic tree of all SAR11 genomes available, based on concatenated shared genes, with the relative positions of all genospecies as well as different subclasses defined so far (33). In order to avoid possible coverage biases, we analyzed the effect on the *pN/pS* ratio using a range from 100,000 to 1 million reads per genome, providing a range from ca. 10× to 100× coverage. We used as references three SAR11 genomospecies in three metagenomes. Table S1 shows that although the average percentage of polymorphic sites (PPS) per gene increased with coverage, *pN/pS* values remained constant, indicating that the effect of coverage on this parameter is negligible. Given the enormous diversity and the uneven recruitment coverage within SAR11 populations, mapped reads were subsampled to 1 million reads per genome and sample. This way, the number of reads was always the same, regardless of the depth coverage of the genome in the sample.

10.1128/mSystems.00605-20.1 FIG S1 Maximum likelihood phylogenomic tree of all SAR11 genomes available to date. (Adapted from reference 33.) Colored dots next to the genome identifier indicate the origin of the genome, that is, metagenome-assembled genome (MAG) (red), single-cell genome (SAG) (blue), or pure culture (yellow). Branches of the tree are colored according to a previous classification (28). Sequences were grouped within subclades and genomospecies (black or white squares). Genomes and genomospecies used in this study are highlighted in red. Download FIG S1, PDF file, 0.1 MB. Copyright © 2020 López-Pérez et al. 2020 López-Pérez et al. This content is distributed under the terms of the Creative Commons Attribution 4.0 International license.

10.1128/mSystems.00605-20.5 TABLE S1 Influence of metagenomic coverage on the rates of evolutionary dynamics for SAR11 genomospecies. Download Table S1, PDF file, 0.4 MB. Copyright © 2020 López-Pérez et al. 2020 López-Pérez et al. This content is distributed under the terms of the Creative Commons Attribution 4.0 International license.

All analyses were performed with the three most complete genomes of each genomospecies in three different metagenomic samples (Table 1 and Table S2) (only reads with >98% identity to the reference were taken into consideration). The average PPS per gene was always higher than 16%, reaching in some cases up to 40% (Table S2). Despite this broad variation in PPS, *pN/pS* values for most Ia.3 genomospecies were always close to a median of 0.06 (Table 1 and Table S2). Together, the high PPS and low *pN/pS* values suggest strong purifying selection. Along similar lines, the percentage of proteins with a *pN/pS* ratio of >1 was very low (0.5 to 1% of the total). Most of them were hypothetical, regardless of the genome or sample. The Mediterranean Sea genomospecies Ia.3/VII, which showed the highest recruitment values of any Ia.3 genomospecies at any station (33), had only a slightly higher *pN/pS* ratio (0.09).

**TABLE 1:** Rates of evolutionary dynamics, abundances, and recombination for SAR11 genomospecies and other marine microbes

Genomospecies^*a*^ or microbe	Group^*b*^	% polymorphic sites^*c*^	pN	pS	*pN/pS* ratio	Abundance (RPKG)^*d*^	γ/μ ratio	Recombination coverage	Median ANIr (%)^*e*^
Ia.3/I	A	26.30	0.25	4.05	0.06	17.92	20.42	0.61	92.00
Ia.3/IV	A	31.27	0.31	4.98	0.07	22.20	16.81	0.65	91.86
Ia.3/V	A	39.40	0.39	6.95	0.06	64.99	21.00	0.75	94.38
Ia.3/VI	A	34.94	0.37	6.27	0.06	29.10	24.95	0.74	93.00
Ia.3/VII	A	27.16	0.39	4.38	0.09	323.95	21.97	0.67	96.67
Ia.3/VIII	A	37.35	0.44	6.63	0.07	25.21	22.64	0.68	92.55
Ib.1/III	A	33.17	0.45	8.45	0.06	42.54	21.10	0.75	95.05
Ib.2/I	A	45.35	0.87	14.61	0.07	25.81	21.01	0.73	93.33
Ic.1 (bathytype)	A	32.37	0.84	5.53	0.15	11.81	13.58	0.74	93.00
IIIb (freshwater-LD12)	A	16.26	0.48	1.27	0.16	80.32	12.21	0.46	93.07
Alteromonas macleodii AD45	B	8.47	0.19	0.43	0.27	54.67	3.90	0.44	97.33
Erythrobacter citreus LAMA 915	B	4.34	0.13	0.25	0.29	11.83	4.78	0.38	98.00
“*Ca.* Nitrosopelagicus brevis” CN25	B	29.90	1.13	1.80	0.77	128.25	15.85	0.56	95.33
MG-II *Thalassoarchaea*	B	12.33	0.23	0.31	0.39	36.11	20.12	0.39	98.67
Prochlorococcus marinus MED4	B	45.13	1.59	2.15	0.77	180.82	55.59	0.80	95.97
*Synechococcus* sp. CC9902	B	17.06	0.42	0.55	0.47	29.91	8.85	0.37	95.05

a Values are calculated based on the average from the three most complete genomes in three different metagenomic samples.

b A, SAR11 genomospecies; B, reference marine microbes.

c Percentage of polymorphic sites per gene.

d RPKG, reads per kilobase of genome and gigabase of metagenome.

e ANIr, read-based average nucleotide identity.

10.1128/mSystems.00605-20.6 TABLE S2 Rates of evolutionary dynamics, abundance, and recombination for SAR11 genomospecies. Download Table S2, PDF file, 0.4 MB. Copyright © 2020 López-Pérez et al. 2020 López-Pérez et al. This content is distributed under the terms of the Creative Commons Attribution 4.0 International license.

**FIG 1:**
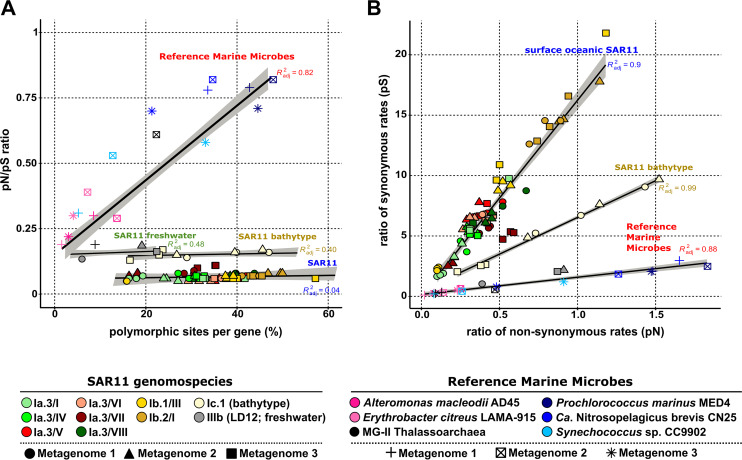
(A) Comparison of the ratio of nonsynonymous to synonymous substitutions (*pN/pS* ratio) (*y* axis) against the percentage of polymorphic sites per gene (*x* axis). (B) Comparison of the ratio of synonymous (*y* axis) to nonsynonymous (*x* axis) rates. Linear regressions and *R*^2^ values are indicated for the different groups: (i) surface oceanic SAR11, (ii) SAR11 bathytype, (iii) SAR11 freshwater, and (iv) reference marine microbes.

Given that within the Ia.3 subclade, there are broad genomic diversities (the minimum pairwise ANI value within each genomospecies was ca. 80%) (33) and distribution patterns across metagenomes, the similarity in these evolutionary parameters (PPS and *pN/pS* ratio) was remarkable. Therefore, we wondered if similar patterns were to be found in other SAR11 subclades. Genomospecies Ib.1/III and Ib.2/I, selected on the grounds of their high abundance values (33, 41), which are also surface oceanic SAR11 genomes, showed similar PPS and *pN/pS* values. However, we observed markedly higher median *pN/pS* values in the bathypelagic subclade Ic (32) and the freshwater clade LD12 (subclade IIIb) (39) (*pN/pS* ratio of ca. 0.16) as well as a decrease in the median PPS in the freshwater subclade IIIb (Fig. 1A, Table 1, and Table S2). Unfortunately, since only one representative (SAG AAA028-C07) of subclade IIIb displayed coverage high enough to carry out the analyses in several metagenomes, we were unable to obtain the averages for three genomes as we did for the other subclades. Interestingly, the *pN/pS* ratios within these other genomospecies remained stable regardless of the PPS values (Fig. 1A), as observed for the surface oceanic SAR11 genomes.

In order to put the PPS and *pN/pS* values of the SAR11 genomospecies into perspective, we applied the same analysis to a collection of bacterial and archaeal groups that share a similar pelagic marine habitat. Within this heterogeneous group, we selected microbes with different population densities (abundances) and ecological strategies, autotrophs and (photo)heterotrophs (8, 42), including copiotrophic bloomers (large genomes) (Table 1 and Table S3). Here, we refer to this set as reference marine microbes (RMM). In all cases, the *pN/pS* values were higher than those detected in SAR11 (Fig. 1A, Table 1, and Table S3). The cyanobacterium Prochlorococcus marinus MED4, representative of the high-light-adapted ecotype, and the thaumarchaeal genome of “*Candidatus* Nitrosopelagicus brevis” CN25 had similar average PPS values within the range of those obtained for SAR11 genomospecies, but the *pN/pS* values were more than 10 times higher (0.77) (Fig. 1A, Table 1, and Table S3). The two copiotrophic heterotrophs Alteromonas macleodii and Erythrobacter citreus (16, 43) had lower PPS and *pN/pS* ratio (ca. 0.28) values, although the *pN/pS* ratios were again higher than those for SAR11 (Table 1). In the case of these opportunistic bacteria, they probably have bloom and crash cycles (42, 44, 45) starting from a few cells and generate more homogeneous populations with lower diversity (see below).

10.1128/mSystems.00605-20.7 TABLE S3 Rates of evolutionary dynamics, abundance, and recombination for reference marine microbes. Download Table S3, PDF file, 0.4 MB. Copyright © 2020 López-Pérez et al. 2020 López-Pérez et al. This content is distributed under the terms of the Creative Commons Attribution 4.0 International license.

This analysis suggests that among SAR11 genomospecies, *pN/pS* values do not vary across a broad range of PPS values (Fig. 1A), while among RMM genomes (Fig. 1A), we observed a clear-cut positive relationship (*R*^2^, 0.82) between PPS and *pN/pS* values. To analyze this phenomenon in depth, we evaluated the relative contributions of synonymous and nonsynonymous mutations to the overall *pN/pS* ratio. These results showed three distinct patterns with an almost linear correlation (*R*^2^, 0.88 to 0.99), where the fraction of synonymous replacements (pS) seemed to be the differential factor (Fig. 1B). Thus, in surface oceanic SAR11 genomospecies, we observed a higher proportion of synonymous replacements, with values up to 10 times higher than for nonsynonymous replacements (pS values of up to 20) (Fig. 1B, Table 1, and Table S2), while in RMM and freshwater SAR11 genomes, none of them had values of pS of >3 (Fig. 1, Table 1, and Table S3). This relationship was less pronounced for the SAR11 bathytype (Ic.1), which displayed a trend resembling those observed for the surface SAR11 and RMM genomes (Fig. 1B).

Given the uniqueness of these parameters in SAR11 epipelagic genomospecies, we examined whether this phenomenon was a consequence of their high population densities (28). For that reason, we calculated population densities by applying metagenomic fragment recruitment analysis, normalized by the number of reads per kilobase of genome and gigabase of metagenome (RPKG). The results showed no correlation between pS and RPKG values, neither among genomospecies nor among different genomes within the same genomospecies (Fig. S2). For instance, the genomospecies Ia.3/VII genomes, which had the highest recruitment values of all groups (average of 320 RPKG), had a pS value of <6, while genomospecies Ib.2/I, with much lower relative abundance values (average of 26 RPKG), always had pS values of >10 (Fig. S2 and Table 1). In contrast, for RMM, we found that an increase in the relative abundance was associated with increasing ratios of both synonymous and nonsynonymous substitutions (Table 1, Table S3, and Fig. S2).

10.1128/mSystems.00605-20.2 FIG S2 Comparison of the ratios of synonymous substitution rates (pS) (*y* axis) against the logarithm of the abundance (measured in RPKG) (*x* axis). Download FIG S2, PDF file, 0.04 MB. Copyright © 2020 López-Pérez et al. 2020 López-Pérez et al. This content is distributed under the terms of the Creative Commons Attribution 4.0 International license.

**FIG 2:**
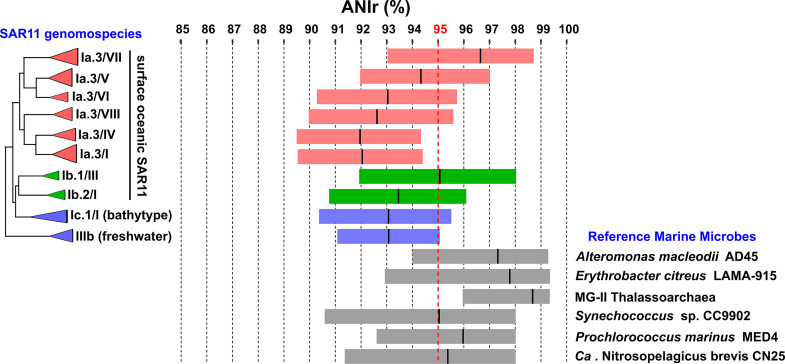
Box plot indicating the average nucleotide identity based on metagenomic reads (ANIr) among SAR11 subclades and some reference marine microbes. Boxes in red represent different genomospecies belonging to the Ia.3 subclade, while boxes in green belong to the Ib subclade. Boxes in blue represent two different SAR11 ecotypes, one collected from bathypelagic waters (subclade Ic) and the other from freshwater samples (subclade IIIb). The red dotted line indicates the species threshold (95% identity). A maximum likelihood phylogenomic tree of the SAR11 genomospecies is shown on the left.

Next, we sought to delve deeper into the intrapopulation sequence diversity within each genomospecies using metagenomic reads to calculate the read-based average nucleotide identity (ANIr). All but one of the SAR11 genomospecies were characterized by ANIr values well below 95%, which is generally accepted as the species threshold (46) (Fig. 2 and Table 1). Only the genomospecies Ia.3/VII, with a preferential Mediterranean occurrence (33), had a median ANIr value of 96.7%. These data could reflect a more recent divergence associated with a more modern habitat (the Mediterranean Messinian salinity crisis [47] happened only 6 million years ago). The acquisition of a set of genes involved in phosphonate utilization in the flexible genome has been suggested to be an essential part of the success of this genomospecies in the phosphate-depleted Mediterranean Sea (33). Meanwhile, RMM genomes showed lower intrapopulation sequence diversity, with ANIr values never below 95% (Fig. 2 and Table 1). In agreement with their ecological strategy (bloomers), A. macleodii and E. citreus showed ANIr values above 97%. As mentioned above, lower intrapopulation sequence diversity values might indicate more recent clonal sweeps (18).

### Homologous recombination.

**FIG 3:**
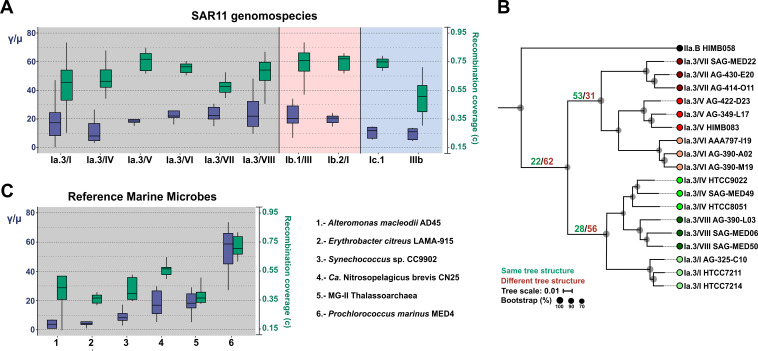
(A) Box plot showing the ratio of recombination to mutation (γ/μ) (left *y* axis) (blue boxes) and the fraction of the sample diversity (*c*) that results from recombination events (right *y* axis) (green boxes). (B) Phylogenomic tree of the Ia.3 subclade using 232 shared proteins in common and considering only the three most complete genomes per genomospecies. The isolated genome of HIMB058, which belongs to a distant subclade, was used as an outgroup. Numbers located in the three most ancient branches and in green or red indicate the number of proteins that, after an individual phylogenetic analysis, produced or failed to rescue the same topology, respectively. Bootstrap values are indicated as black circles on the nodes. (C) Similar to panel A but with some reference marine microbes.

To discriminate between single nucleotide polymorphisms introduced by mutation and those introduced by genetic exchange (homologous recombination) (48), we computed the relative rate of recombination to mutation (γ/μ) (48) using, as described above, the three most complete genomes in the three metagenomic samples of maximum recruitment across the same SAR11 genomospecies. Mean estimates of the γ/μ ratio were similar for all the genomospecies of the Ia.3 subclade, Ib.1/III, and Ib.2/I (ca. 20), with the only exception being genomospecies Ia.3/IV, a genomospecies associated with the deep chlorophyll maximum (33), which had a slightly lower ratio (ca. 16.8) (Fig. 3A and Table 1). Therefore, for all surface genomospecies, recombination-driven nucleotide replacements were much more frequent than nucleotide mutations. However, the γ/μ values for the freshwater subclade IIIb and the marine bathytype Ic.1 were half of those observed for the surface oceanic clade (Fig. 3A and Table 1). These results had been previously reported using single-cell genomics, thus corroborating the reliability of both methods (34). A parameter that might affect the recombination rate could be the cell density that can be estimated by recruitment (RPKG). A lower population density could lead to a reduction in the recombination rate as in the case of the deep-ocean SAR11 bathytype (Table 1). We also measured the fraction of genomes in the samples that have undergone recombination (“*c*” [recombination coverage]), which ranges from 0 to 1, taking 0 as the population that has evolved without recombination (48). These data reveal that within Ia.3 genomospecies, between 0.60 and 0.75 of the reference genomes had undergone recombination (Fig. 3A). Similar values were obtained for the other marine SAR11 groups analyzed. On the other hand, *c* dropped to 0.46 for the freshwater subclade IIIb (Fig. 3A and Table 1). To double-check this high level of recombination detected for the Ia.3 genomospecies, we generated individual phylogenetic trees for 84 core genes and compared them against the consensus tree generated by a concatenation of all genes present in all genomes (232 genes). The results (Fig. 3B) supported the highly recombinogenic nature of SAR11 populations (37) and the conclusion drawn from metagenomic data.

The opportunistic bloomers *A. macleodii* and *E. citreus* showed γ/μ values of ca. 4, similar to those obtained using phylogenomic comparisons (49). Significantly, higher γ/μ values were found for *Synechococcus*, within the range of those detected in freshwater SAR11 genomospecies IIIb and bathytype Ic representatives (Fig. 3C and Table 1). In addition, the two archaeal genomes analyzed (from the phyla *Euryarchaeota* and *Thaumarchaeota*) showed γ/μ values comparable to those of SAR11 (ca. 20). Finally, *Prochlorococcus* had γ/μ values close to three times those of SAR11 (ca. 60), although the recombination coverage was similar (Fig. 3 and Table 1). The *c* values were lower for all other microbes, ranging from 0.33 to 0.58 of the reference genome (Fig. 3C, Table 1, and Table S3). We also compared these SAR11 parameters (γ/μ ratio and *c*) with those obtained in some pathogenic microbes using the same methodology (48). Mycobacterium abscessus and Pseudomonas aeruginosa (known to be highly recombinogenic [50, 51]), with the highest recombination values (13 and 11, respectively), had γ/μ values close to half of those detected in the surface oceanic SAR11 clades.

Although the specific mechanisms of gene transfer in SAR11 populations are unknown, it seems to be clear that these microbes exchange parts of their genome with remarkable frequency. It has been suggested that SAR11 can take up DNA from the environment due to the presence of DNA uptake and competence genes (52). Furthermore, we found a prophage inserted in a tRNA-Val in the SAG-MED28 genome (Fig. S3) that clustered with several viral sequences recovered from a metagenomic sample from the Mediterranean deep chlorophyll maximum (53). While this SAG is a member of another subclade (IIa.B), this showed that transduction occurs among SAR11 clades as proposed previously (54).

10.1128/mSystems.00605-20.3 FIG S3 Genomic fragments between two SAR11 single-amplified genomes belonging to the genomospecies IIa.B and a viral metagenomic fosmid collected from the deep chlorophyll maximum in the Western Mediterranean Sea. Genes in green and blue indicate a bacterial or viral origin, respectively. Download FIG S3, PDF file, 0.02 MB. Copyright © 2020 López-Pérez et al. 2020 López-Pérez et al. This content is distributed under the terms of the Creative Commons Attribution 4.0 International license.

### Environmentally persistent clones.

Despite the remarkably high intrapopulation diversity in SAR11, the increased genomic diversity in public data sets has led to the discovery of two pairs of nearly identical genomes from different samples and years, i.e., evidence of environmentally persistent clones. Specifically, HTCC7217 and HTCC7211, belonging to the genomospecies Ia.3/I, were isolated from the Bermuda-Atlantic Time Series (BATS) site in 2006 (55). While these genomes had a divergence of 95% ANI, the genomes from HTCC7217 and the single-amplified genome AG-414-C04, which were retrieved from the same region but 4 years apart, presented an ANI of 99.8% (Fig. S4). Along these lines, we found another single-amplified genome (SAG-MED25 [33]) that was nearly identical to the other BATS isolate, HTCC7211 (99.8% ANI), in the Mediterranean Sea, 9 years later (Fig. S4). The coverage of the single-amplified genome on the pure-culture genomes was in both cases more than 70%. Furthermore, the gene contents of the flexible regions found to be drastically different between HTCC7217 and HTCC7211, including the previously identified hypervariable region 2 (HVR2) (56), were also conserved in these nearly identical genomes rescued much later (Fig. S4). These results suggest that there are SAR11 lineages with high persistence and minimal genomic variation within the available time frames (years apart).

10.1128/mSystems.00605-20.4 FIG S4 Pairwise comparison among four SAR11 genomes belonging to the Ia.3/I genomospecies. The date and origin of isolation are shown for each strain. Arrows indicate the position of the genomic islands. ANI, average nucleotide identity; Cov, percentage of the genome aligned. Download FIG S4, PDF file, 0.02 MB. Copyright © 2020 López-Pérez et al. 2020 López-Pérez et al. This content is distributed under the terms of the Creative Commons Attribution 4.0 International license.

## DISCUSSION

**FIG 4:**
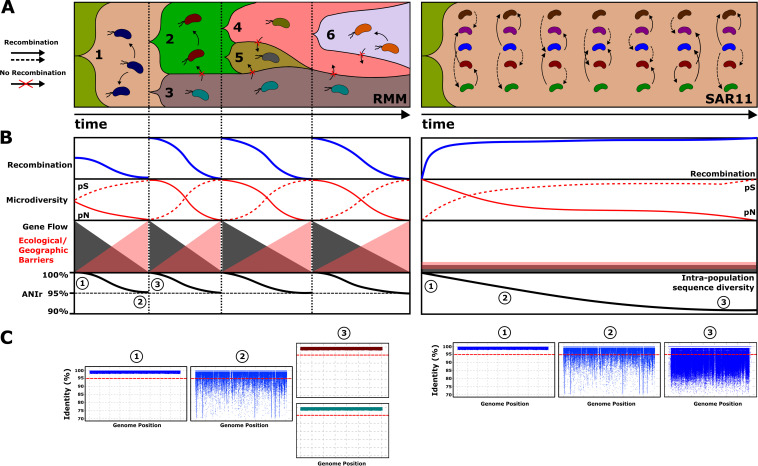
Evolutionary model for reference marine microbes (RMM) and SAR11 populations. RMM evolution is characterized by “hard” selective sweeps that reduce genomic diversity, and only a single adaptive lineage arises from the population. However, SAR11 evolution is characterized by “soft” sweeps, where multiple beneficial mutations contribute to an adaptive substitution, which are dispersed by the population, contributing to an increase in genetic diversity. (A) Relative abundances of the populations (*y* axis) and evolution over time (*x* axis). (B) Dynamics of different evolutionary parameters over time, where each vertical dotted line differentiates different diversification events in RMM, while in SAR11 populations, the same structure is maintained over time. (C) Recruitment plot showing intrapopulation sequence diversity. The red dashed line indicates the species threshold (95%). Different colors indicate different populations.

Understanding the high genomic level of heterogeneity within marine prokaryotic populations has been a challenge for microbiologists in recent years (57). In asexual microorganisms, one of the possible scenarios proposed to explain such diversity is the presence of several clonal subpopulations (or ecotypes) with different ecological adaptations to discrete niches, which generates barriers and promotes the decrease of recombination between them (58). Another possibility is that these subpopulations occupy the same niche, and the overall diversity is provided, mainly, by high recombination rates, preventing clonal sweeps in a “quasisexual” manner (59). Multiple evolutionary models have been proposed to explain the diversification of distinct lineages within a population (40, 60, 61); however, the identification of many of these patterns in natural populations is something that has not been elucidated so far. Using a metagenomics approach, we have studied the ecological and evolutionary processes of natural populations of SAR11. Our results are in agreement with evolutionary dynamics of some SAR11 representatives consistent with quasisexual evolution, as has been described for cyanobacterial biofilms (59), where high recombination rates between closely and distantly related lineages promote the homogenization of the populations, leading to a stable population that may remain unchanged (but with high intrapopulation diversity) for extended periods (Fig. 4A and B). The cohesiveness of the core genome driven by homologous recombination has also been explained by mathematical models (62). Recently, a similar evolutionary regime has been characterized using a computational model and defined the concept of “metastable” populations (40). In addition, we observed an accumulation of synonymous replacements that, combined with the high intrapopulation sequence diversity, suggests an evolutionary scenario in which nonsynonymous mutations probably have been purged over time since purifying selection cannot act at short time scales (Fig. 4B). Therefore, this could support the idea of a very ancient divergence of SAR11 populations. The presence of high genomic diversity within each population (genomospecies) might be maintained by negative density-dependent selection by viruses (kill the winner) as predicted by the constant-diversity model (14). This is reflected in the linear recruitment plots, where the threshold is located above 80% identity, and by the higher intrapopulation sequence diversity (ANIr of <95%) showing less clonal populations (Fig. 4C). This high genomic diversity of SAR11 genomospecies might provide the population with better flexibility to adjust to environmental oscillations (25), such as a greater affinity for certain micronutrients with patchy distributions near the nutrient-depleted surface. Within the broad environmental niche (surface waters of oceans around the world), if there are no barriers to gene flow, all SAR11 populations could be able to remain at a basal level of abundance provided that their extinction is prevented and multiple beneficial mutations spread throughout the population at the same time (“soft sweeps”) (60, 63). Interestingly, the adaptation of SAR11 genomospecies to other environments in which their populations are less dense, such as the bathypelagic ocean, makes their genomic diversification more similar to that of the RMM with higher, density-dependent *pN/pS* ratios.

In comparison with a study conducted by Delmont and collaborators (25), we have identified far fewer nonsynonymous variables. This can be attributed to differences in mapping stringencies, coverage cutoffs, algorithms identifying variants, and, finally, the portions of the genomes being considered.

In contrast, the selected RMM (regardless of their ecological strategy) appear to adjust more closely to the ecotype model (57, 64). A higher relative abundance (more read recruitment) is positively correlated with higher PPS values, and these are correlated with a higher *pN/pS* ratio (nonsynonymous mutations that have not yet been purged) (Fig. 4B). This phenomenon could be driven by an earlier stage of ecological, geographic, or behavioral processes of diversification (65). Unlike SAR11 evolution (soft sweep), successive “hard” selective sweeps seem to dominate the evolution of these other marine microbes, where part of the genomic diversity is purged from the population by the rise of a few adaptive lineages (66). There is also the possibility that higher growth rates (or sporadic growth rates in the case of bloomers) generate mutation rates that selection cannot purge before the next crash of the population. These demographic patterns would also lead to lower intrapopulation sequence diversity (ANIr of >95%) (Fig. 4C). Overall, the results obtained in this study improve our understanding of the evolutionary processes behind marine microbial populations and, in particular, shed light on the extraordinary success of SAR11. Our evolutionary model and metagenomic approaches are broadly applicable to examine ecological and evolutionary patterns in natural populations in other environments.

## MATERIALS AND METHODS

### Genome retrieval from public data sets.

Genomes belonging to the SAR11 clade (marine and freshwater) were downloaded from the NCBI database and phylogenomically classified in previous work (33). In addition, some representatives of well-known marine microbes were also downloaded from the NCBI: Alteromonas macleodii AD45 (NCBI accession number GCF_000300185.1), Erythrobacter citreus LAMA-915 (NCBI accession number GCA_001235865.1), *Synechococcus* sp. strain CC9902 (NCBI accession number GCA_000012505.1), “*Ca*. Nitrosopelagicus brevis” CN25 (NCBI accession number GCA_000812185.1), MG-II *Thalassoarchaea* (BioSample accession number SAMN02954236), and Prochlorococcus marinus MED4 (NCBI accession number GCA_000011465.1). The general features of these genomes are shown in Table S4 in the supplemental material. For each genome, coding DNA sequences were predicted using Prodigal (67). tRNA and rRNA genes were predicted using tRNAscan-SE (68), ssu-align (69), and meta-rna (70). To infer the function, predicted protein sequences were compared against NCBI NR databases using DIAMOND (71) and against COG (72) and TIGFRAM (73) using HMMscan (74).

10.1128/mSystems.00605-20.8 TABLE S4 List of all genome sequences used in this work. The table shows the genome completeness (percent) and degree of contamination (percent) computed using CheckM. Download Table S4, PDF file, 0.2 MB. Copyright © 2020 López-Pérez et al. 2020 López-Pérez et al. This content is distributed under the terms of the Creative Commons Attribution 4.0 International license.

### Metagenomic fragment recruitment.

Several metagenomic data sets were used to recruit reads against several SAR11 subclades (including the freshwater LD12 and the marine bathypelagic ecotypes) and some reference marine microbes (see above). Briefly, raw reads from the *Tara* Oceans (3) and GEOTRACES (31) expeditions and a metagenomic data set collected at different depths, years, and seasons from the Mediterranean Sea (8, 75) were downloaded from the ENA and NCBI databases (BioProject accession numbers PRJEB1787, PRJNA385854, PRJNA352798, and PRJNA257723). In addition, we performed recruitment analyses of several freshwater metagenomes downloaded from the JGI database (https://img.jgi.doe.gov/).

To avoid an overestimation of genome abundances (33) in the samples, the complete ribosomal operon gene cluster was manually removed from each genome sequence prior to recruitment. Metagenomic reads were trimmed using Trimmomatic v0.36 (76). Only reads with a Phred score of ≥30, that were ≥50 bp long, and that had no ambiguous bases (N’s) were kept. These high-quality trimmed metagenomic reads were then aligned using BLASTN (77), using a cutoff of 98% nucleotide identity and an alignment length of ≥50 nucleotides. They were used to compute the RPKG (reads recruited per kilobase of genome and per gigabase of metagenome) values, which provide a normalized number comparable across various metagenomes. Since different data sets with different read lengths (Illumina HiSeq 2×100 bp and 2×150 bp) were used for recruitment, each metagenome was also normalized, dividing the size of the database by its average read size.

### Pairwise comparison between genomes and environmental metagenomic reads.

The average nucleotide identity (ANI) between a pair of genomes was calculated using JSpecies software with default parameters (78). Meanwhile, the average nucleotide identity of metagenomic short reads (ANIr) was calculated by recruiting high-quality trimmed metagenomic reads (see above) against reference genomes using BLASTN (77), with a cutoff of 80% nucleotide identity and an alignment length of ≥50 nucleotides.

### Recombination rates among groups.

High-quality trimmed metagenomic reads were aligned against individual genomes using the Bowtie2 -sensitive-local mode (79). In more detail, for each SAR11 genomospecies, three genomes were used to align reads from three metagenomes. Conversely, for the freshwater LD12 clade and the other marine representative genomes, only one genome was used to align reads from three metagenomes. The resulting SAM files were converted and sorted into BAM files using SAMtools (80) and used to carry out the analysis of the rates of recombination among groups. In a first approach, we applied mcorr software (https://github.com/kussell-lab/mcorr) (48) to infer the parameters of homologous recombination within *in situ* samples, that is, the rate of recombination to mutation (γ/μ) and the fraction of the recombination coverage (*c*) that results from recombination events. As described previously (48), a *c* value of 0 indicates clonal evolution, whereas if the value reaches 1, the microbe has recombined nearly its whole genome.

In another approach, we analyzed in more detail the high rate of recombination within the Ia.3 subclade (genomospecies I, IV, V, VI, VII, and VIII) (33) by phylogenetic analyses. To do that, encoded proteins were clustered using cd-hit (81), with identity and alignment thresholds of 70% identity and 80% length, respectively. Only clusters with one protein per genome were considered. In the end, 84 shared proteins among genomes were selected and phylogenetically studied individually. Individual sets of proteins were aligned with muscle (82), and a maximum likelihood phylogenetic tree was constructed using iq-tree (83) with the following parameters: Jones-Taylor-Thornton model, five discrete rate gamma categories, 1,000 ultrafast bootstrap approximations, and elimination of positions with <80% site coverage. Next, the resulting topologies were compared to the phylogenomic tree obtained by using all shared proteins reported previously (33).

### Microdiversity.

To estimate mutational frequencies, raw reads were mapped to assembled genomes using Bowtie2 (79). Following read mapping, the generated bam files were downsampled to 1 million reads per genome. This step was performed to avoid that differences in genome coverage affected the subsequent results. Next, the subsampled BAM files were analyzed through Diversitools (http://josephhughes.github.io/DiversiTools/) to obtain counts of synonymous and nonsynonymous mutations in each protein, from each genome in each tested metagenome sample. We considered valid only those codon mutations that were detected at least four times, in at least 0.1% of the mapped reads, with a coverage equal to or above 5×. The frequencies of mutations that passed the above-mentioned criteria were used as the input to calculate *pN/pS* ratios and the percentage of polymorphic sites, as previously described (84). We calculated the *pN/pS* ratio using a range from 100,000 to 1 million reads per genome in order to analyze the coverage bias. Finally, 1 million reads per genome and sample were used for the analysis to normalize the values.
