# Supplementary figures and images for: The Evolutionary Success of the Marine Bacterium SAR11 Analyzed through a Metagenomic Perspective
Source: mSystems. 2020 Oct 6;5(5):e00605-20. doi: 10.1128/mSystems.00605-20 (PMC7542561; doi:10.1128/mSystems.00605-20)

- ## ● Pure culture

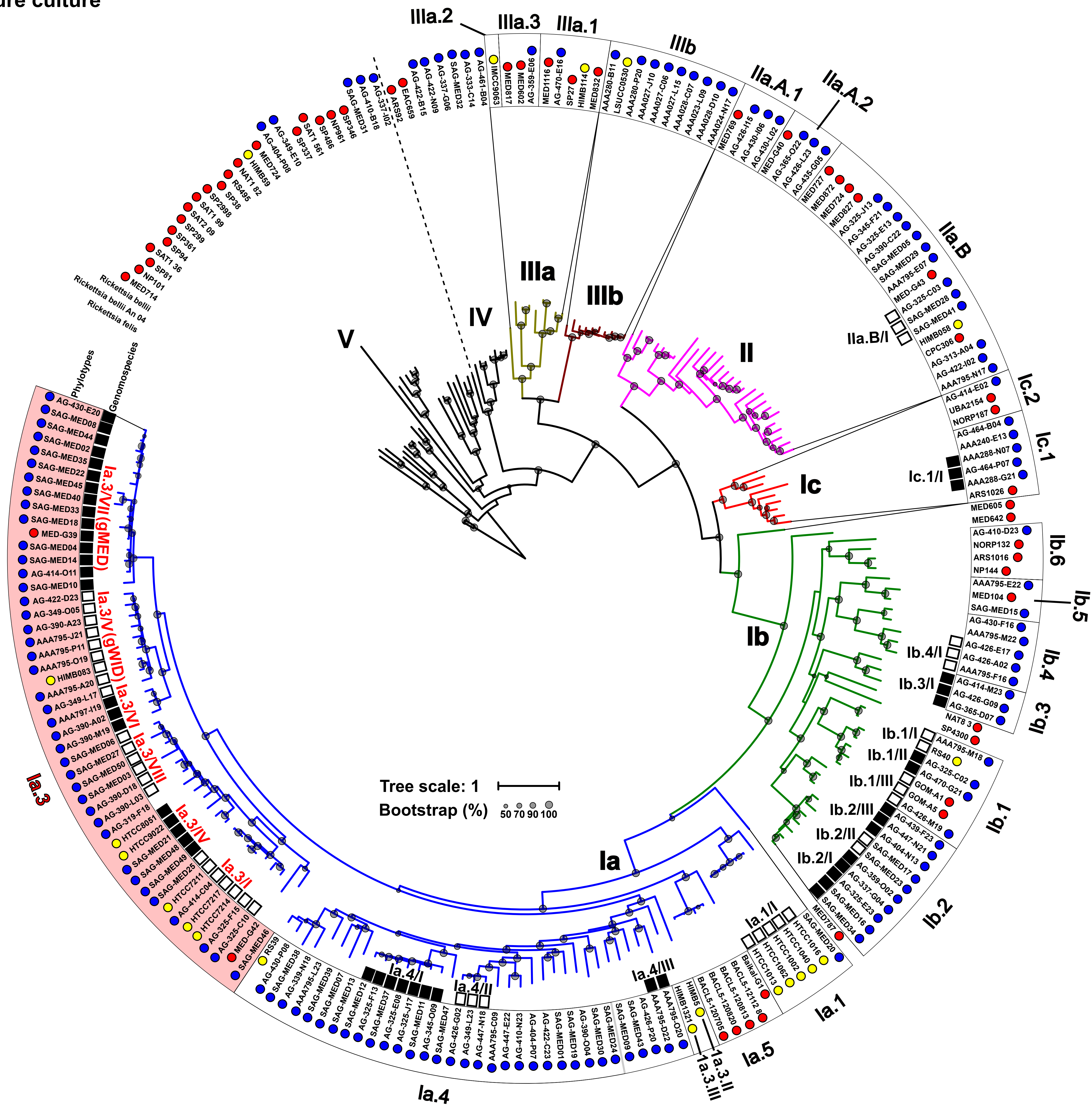

Supplement: FIG S1 [file mSystems.00605-20-sf001.pdf]

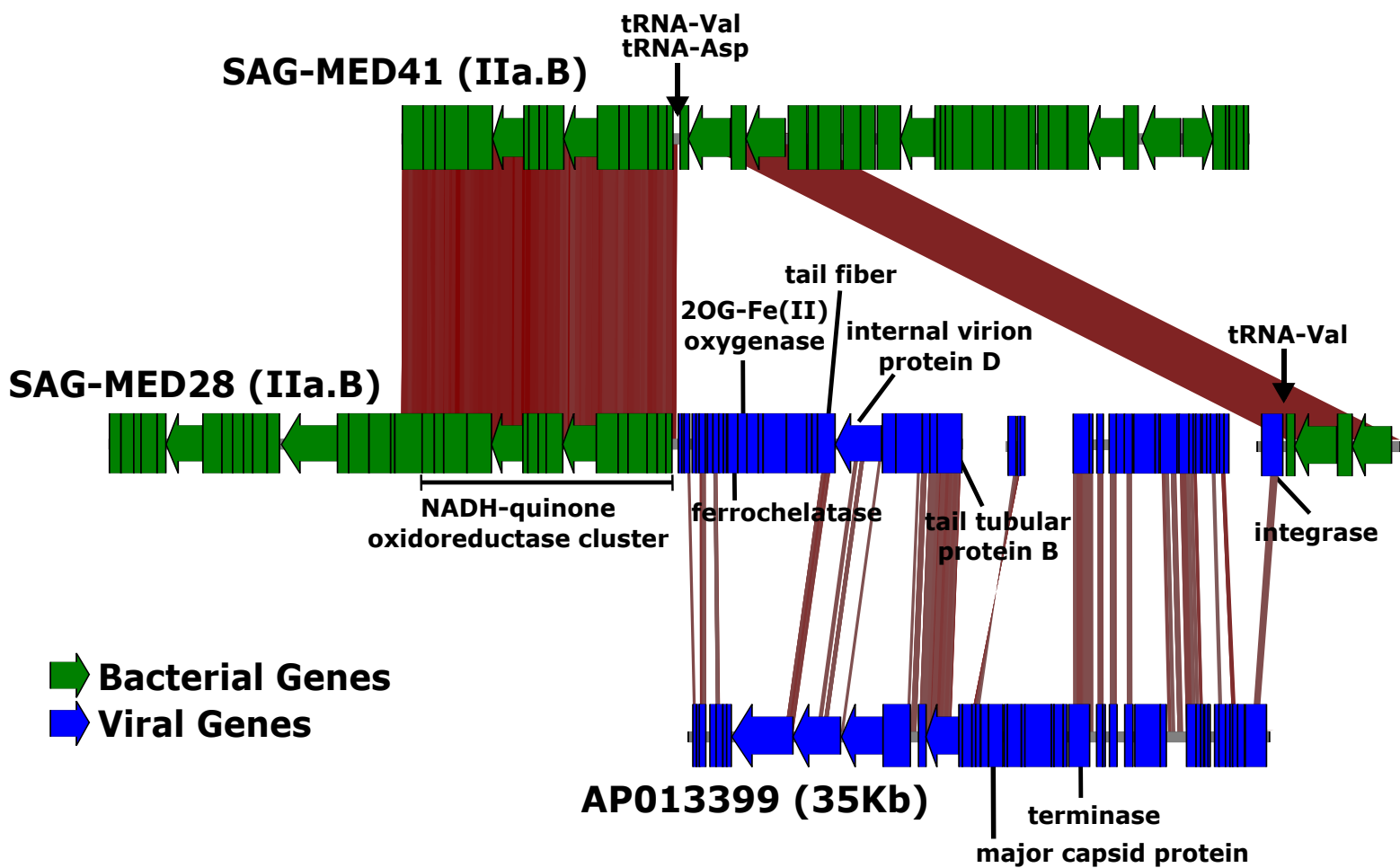

Supplement: FIG S3 [file mSystems.00605-20-sf003.pdf]

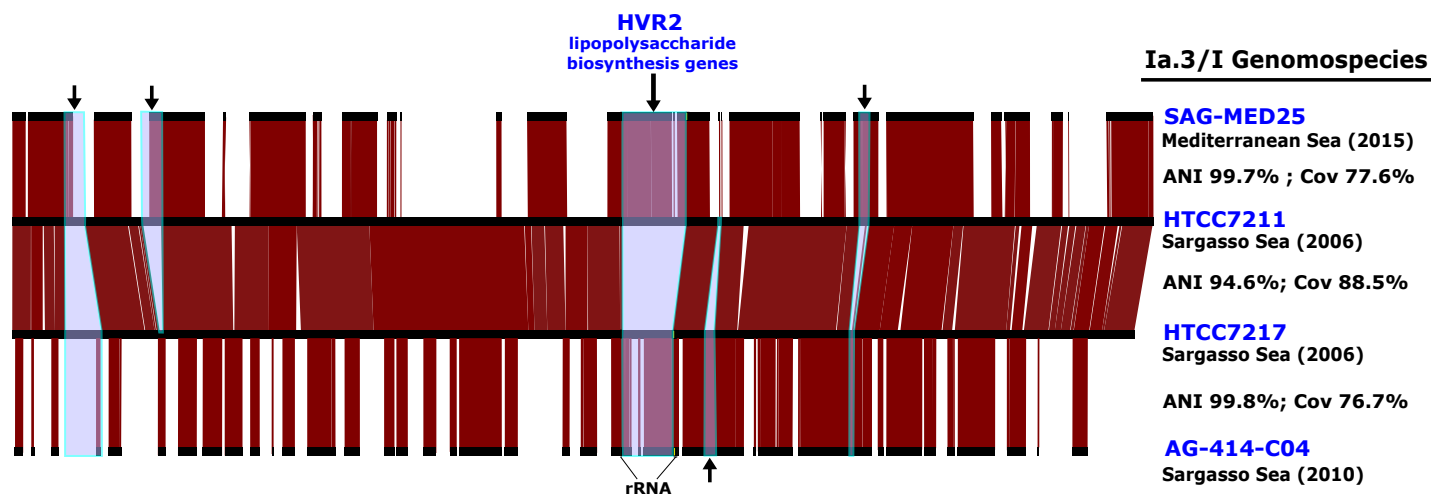

Supplement: FIG S4 [file mSystems.00605-20-sf004.pdf]
