# Supplementary material for: The Evolutionary Success of the Marine Bacterium SAR11 Analyzed through a Metagenomic Perspective
Source: mSystems. 2020 Oct 6;5(5):e00605-20. doi: 10.1128/mSystems.00605-20 (PMC7542561; doi:10.1128/mSystems.00605-20)
Supplement: TABLE S1 [file mSystems.00605-20-st001.pdf]

SAR11 Genomospecies[1]	#of sequences (estimated coverage)	Polymorphic sites[2] (%)	pN	pS	pN/pS ratio
Ia.3/V	100K (10X)	1.30	0.01	0.27	0.08
	250K (25X)	4.10	0.07	1.18	0.07
	500K (50X)	7.40	0.19	2.80	0.07
	**1M (100X)**	**39.40**	**0.39**	**6.95**	**0.06**
Ia.3/VII	100K (10X)	1.50	0.02	0.32	0.11
	250K (25X)	3.20	0.08	0.96	0.11
	500K (50X)	5.70	0.20	2.30	0.10
	**1M (100X)**	**27.16**	**0.39**	**4.38**	**0.09**
Ib.2/I	100K (10X)	1.50	0.02	0.34	0.09
	250K (25X)	4.40	0.10	1.30	0.08
	500K (50X)	7.50	0.23	3.16	0.08
	**1M (100X)**	**45.35**	**0.87**	**14.61**	**0.07**

[1]Values are calculated based on the average of the most complete genome in three different metagenomic samples

[2]Percentage of polymorphic sites per gene
