# Supplementary material for: The Evolutionary Success of the Marine Bacterium SAR11 Analyzed through a Metagenomic Perspective
Source: mSystems. 2020 Oct 6;5(5):e00605-20. doi: 10.1128/mSystems.00605-20 (PMC7542561; doi:10.1128/mSystems.00605-20)
Supplement: TABLE S2 [file mSystems.00605-20-st002.pdf]

Genome	Genomospecies	Metagenome	Biosample	Bioproject	Depth[1]	Isolation source	Polymorphic sites[2] (%)	pN	pS	pN/pS ratio	Abundance (RPKG[3])	γ/μ	Recombination coverage
AG-325-C10	Ia.3/I	ERR598978 (TARA 102)	SAMEA2622173	PRJEB1787	SRF (5m)	South Pacific Ocean	41.77	0.56	9.69	0.06	24.04	18.92	0.63
AG-325-C10	Ia.3/I	ERR315857 (TARA 007)	SAMEA2591057	PRJEB1788	SRF (5m)	Mediterranean Sea	27.12	0.26	4.30	0.05	11.80	15.16	0.66
AG-325-C10	Ia.3/I	SRR1539383 (MedDCM-JUL2012)	SAMN02954012	PRJNA257723	DCM (75m)	Mediterranean Sea	16.34	0.10	1.59	0.06	8.52	21.51	0.53
HTCC7211	Ia.3/I	ERR598978 (TARA 102)	SAMEA2622173	PRJEB1787	SRF (5m)	South Pacific Ocean	33.02	0.31	5.55	0.06	18.92	19.24	0.72
HTCC7211	Ia.3/I	ERR315857 (TARA 007)	SAMEA2591057	PRJEB1788	SRF (5m)	Mediterranean Sea	24.58	0.21	3.16	0.06	29.44	21.51	0.53
HTCC7211	Ia.3/I	SRR1539383 (MedDCM-JUL2012)	SAMN02954012	PRJNA257723	DCM (75m)	Mediterranean Sea	19.38	0.14	1.84	0.07	14.75	20.86	0.54
HTCC7214	Ia.3/I	ERR598978 (TARA 102)	SAMEA2622173	PRJEB1787	SRF (5m)	South Pacific Ocean	32.57	0.31	5.41	0.06	23.13	17.85	0.62
HTCC7214	Ia.3/I	ERR315857 (TARA 007)	SAMEA2591057	PRJEB1788	SRF (5m)	Mediterranean Sea	23.96	0.21	3.21	0.06	19.06	23.57	0.63
HTCC7214	Ia.3/I	SRR1539383 (MedDCM-JUL2012)	SAMN02954012	PRJNA257723	DCM (75m)	Mediterranean Sea	17.96	0.12	1.70	0.06	11.66	25.17	0.59
HTCC8051	Ia.3/IV	ERR598942 (TARA 133)	SAMEA2623135	PRJEB1787	DCM (45m)	North Pacific Ocean	33.10	0.30	5.24	0.06	24.69	11.44	0.65
HTCC8051	Ia.3/IV	ERR599052 (TARA 133)	SAMEA2623116	PRJEB1787	SRF (5m)	North Pacific Ocean	32.83	0.31	5.08	0.07	26.22	12.52	0.64
HTCC8051	Ia.3/IV	SRR1539383 (MedDCM-JUL2012)	SAMN02954012	PRJNA257723	DCM (75m)	Mediterranean Sea	32.88	0.33	5.06	0.07	10.15	18.20	0.65
HTCC9022	Ia.3/IV	ERR598942 (TARA 133)	SAMEA2623135	PRJEB1787	DCM (45m)	North Pacific Ocean	32.09	0.29	5.67	0.06	24.55	11.10	0.61
HTCC9022	Ia.3/IV	ERR599052 (TARA 133)	SAMEA2623116	PRJEB1787	SRF (5m)	North Pacific Ocean	31.99	0.30	5.55	0.06	15.71	13.13	0.62
HTCC9022	Ia.3/IV	SRR1539383 (MedDCM-JUL2012)	SAMN02954012	PRJNA257723	DCM (75m)	Mediterranean Sea	29.50	0.25	4.52	0.06	25.17	27.93	0.57
SAG-MED49	Ia.3/IV	ERR598942 (TARA_133)	SAMEA2623135	PRJEB1787	DCM (45m)	North Pacific Ocean	30.77	0.36	5.10	0.07	22.61	13.77	0.73
SAG-MED49	Ia.3/IV	ERR599052 (TARA 133)	SAMEA2623116	PRJEB1787	SRF (5m)	North Pacific Ocean	31.14	0.36	4.96	0.07	36.59	17.79	0.70
SAG-MED49	Ia.3/IV	SRR1539383 (MedDCM-JUL2012)	SAMN02954012	PRJNA257723	DCM (75m)	Mediterranean Sea	27.12	0.29	3.63	0.08	14.15	25.40	0.65
AAA795-A20	Ia.3/V	ERR599158 (TARA 038)	SAMEA2620000	PRJEB1787	SRF (5m)	Indian Ocean	44.35	0.52	7.76	0.07	79.80	20.64	0.80
AAA795-A20	Ia.3/V	ERR599029 (TARA 141)	SAMEA2623446	PRJEB1787	SRF (5m)	North Atlantic Ocean	39.72	0.37	7.72	0.06	52.53	19.97	0.72
AAA795-A20	Ia.3/V	ERR598959 (TARA 034)	SAMEA2619879	PRJEB1787	SRF (5m)	Red Sea	41.44	0.42	7.62	0.06	87.88	16.88	0.76
AG-422-D23	Ia.3/V	ERR599158 (TARA_038)	SAMEA2620000	PRJEB1787	SRF (5m)	Indian Ocean	40.28	0.44	6.85	0.07	52.45	20.46	0.78
AG-422-D23	Ia.3/V	ERR598959 (TARA 034)	SAMEA2619879	PRJEB1787	SRF (5m)	Red Sea	37.17	0.37	6.60	0.06	55.41	18.04	0.76
AG-422-D23	Ia.3/V	ERR599029 (TARA 141)	SAMEA2623446	PRJEB1787	SRF (5m)	North Atlantic Ocean	35.16	0.33	6.41	0.05	33.10	26.56	0.71
HIMB083	Ia.3/V	ERR599158 (TARA 038)	SAMEA2620000	PRJEB1787	SRF (5m)	Indian Ocean	41.97	0.42	6.83	0.07	73.51	21.50	0.79
HIMB083	Ia.3/V	ERR598959 (TARA 034)	SAMEA2619879	PRJEB1787	SRF (5m)	Red Sea	38.31	0.34	6.50	0.06	90.69	20.87	0.73
HIMB083	Ia.3/V	ERR599029 (TARA 141)	SAMEA2623446	PRJEB1787	SRF (5m)	North Atlantic Ocean	36.16	0.29	6.28	0.06	59.50	24.12	0.72
AAA797-I19	Ia.3/VI	ERR599029 (TARA_141)	SAMEA2619879	PRJEB1787	SRF (5m)	North Atlantic Ocean	34.33	0.34	6.43	0.05	9.13	32.13	0.71
AAA797-I19	Ia.3/VI	ERR598989 (TARA 137)	SAMEA2623275	PRJEB1787	SRF (5m)	North Pacific Ocean	35.04	0.41	6.19	0.06	53.72	22.65	0.73
AAA797-I19	Ia.3/VI	ERR598966 (TARA 036)	SAMEA2619927	PRJEB1787	SRF (5m)	Indian Ocean	33.02	0.32	5.70	0.06	29.39	22.26	0.71
AG-390-A02	Ia.3/VI	ERR598966 (TARA 036)	SAMEA2619927	PRJEB1787	SRF (5m)	Indian Ocean	36.92	0.40	6.75	0.06	37.22	18.59	0.74
AG-390-A02	Ia.3/VI	ERR598989 (TARA 137)	SAMEA2623275	PRJEB1787	SRF (5m)	North Pacific Ocean	38.13	0.42	6.02	0.07	20.93	25.58	0.80
AG-390-A02	Ia.3/VI	ERR599029 (TARA 141)	SAMEA2619879	PRJEB1787	SRF (5m)	North Atlantic Ocean	29.35	0.27	5.48	0.05	25.45	21.89	0.70
AG-390-M19	Ia.3/VI	ERR598966 (TARA_036)	SAMEA2619927	PRJEB1787	SRF (5m)	Indian Ocean	36.62	0.39	6.72	0.06	40.29	25.71	0.74
AG-390-M19	Ia.3/VI	ERR598989 (TARA 137)	SAMEA2623275	PRJEB1787	SRF (5m)	North Pacific Ocean	39.32	0.46	6.65	0.06	23.36	32.72	0.75
AG-390-M19	Ia.3/VI	ERR599029 (TARA 141)	SAMEA2619879	PRJEB1787	SRF (5m)	North Atlantic Ocean	31.73	0.31	6.48	0.05	22.39	23.00	0.72
AG-414-O11	Ia.3/VII	SRR5007106 (Med-OCT2015-15m)	SAMN05992379	PRJNA352798	SRF (15m)	Mediterranean Sea	30.57	0.44	5.75	0.08	357.15	25.68	0.58
AG-414-O11	Ia.3/VII	SRR5788287 (GEOTRACES GA02S0163)	SAMN07136638	PRJNA385854	SRF (10m)	North Atlantic Ocean	35.23	0.57	5.29	0.11	367.99	19.75	0.54
AG-414-O11	Ia.3/VII	ERR315862 (TARA 030)	SAMEA2591108	PRJEB1787	SRF (5m)	Mediterranean Sea	18.27	0.20	2.68	0.08	528.87	22.61	0.63
AG-430-E20	Ia.3/VII	SRR5007106 (Med-OCT2015-15m)	SAMN05992379	PRJNA352798	SRF (15m)	Mediterranean Sea	30.59	0.43	5.59	0.09	333.36	28.98	0.60
AG-430-E20	Ia.3/VII	SRR5788287 (GEOTRACES GA02S0163)	SAMN07136638	PRJNA385854	SRF (10m)	North Atlantic Ocean	35.38	0.59	5.21	0.11	348.76	23.10	0.57
AG-430-E20	Ia.3/VII	ERR315862 (TARA 030)	SAMEA2591108	PRJEB1787	SRF (5m)	Mediterranean Sea	18.21	0.20	2.67	0.08	544.16	18.69	1.00
SAG-MED22	Ia.3/VII	SRR5007106 (Med-OCT2015-15m)	SAMN05992379	PRJNA352798	SRF (15m)	Mediterranean Sea	28.44	0.37	5.16	0.08	133.33	29.14	0.59
SAG-MED22	Ia.3/VII	SRR5788287 (GEOTRACES GA02S0163)	SAMN07136638	PRJNA385854	SRF (10m)	North Atlantic Ocean	31.36	0.52	4.66	0.10	102.58	17.45	0.55
SAG-MED22	Ia.3/VII	ERR315862 (TARA 030)	SAMEA2591108	PRJEB1787	SRF (5m)	Mediterranean Sea	16.37	0.17	2.45	0.07	199.35	12.32	0.99
AG-390-D18	Ia.3/VIII	Med-OCT2015-60m	SAMN05992382	PRJNA352798	DCM 60m	Mediterranean Sea	39.80	0.52	7.39	0.08	19.61	34.00	0.67
AG-390-D18	Ia.3/VIII	ERR599078 (TARA 152)	SAMEA2623886	PRJEB1787	SRF (5m)	North Atlantic Ocean	39.52	0.45	6.37	0.07	33.23	13.95	0.70
AG-390-D18	Ia.3/VIII	ERR599064 (TARA 093)	SAMEA2621779	PRJEB1787	SRF (5m)	South Pacific Ocean	30.27	0.31	5.29	0.06	24.69	24.31	0.69
AG-390-L03	Ia.3/VIII	Med-OCT2015-60m	SAMN05992382	PRJNA352798	DCM 60m	Mediterranean Sea	39.67	0.52	7.41	0.08	18.68	28.94	0.63
AG-390-L03	Ia.3/VIII	ERR599078 (TARA 152)	SAMEA2623886	PRJEB1787	SRF (5m)	North Atlantic Ocean	38.54	0.41	6.03	0.07	30.30	32.28	0.72
AG-390-L03	Ia.3/VIII	ERR599064 (TARA 093)	SAMEA2621779	PRJEB1787	SRF (5m)	South Pacific Ocean	31.72	0.31	5.69	0.06	22.61	13.70	0.69
SAG-MED50	Ia.3/VIII	SRR5007118 (Med-OCT2015-60m)	SAMN05992382	PRJNA352798	DCM (60m)	Mediterranean Sea	43.85	0.67	8.68	0.08	31.77	20.20	0.52
SAG-MED50	Ia.3/VIII	ERR599078 (TARA 152)	SAMEA2623886	PRJEB1787	SRF (5m)	North Atlantic Ocean	40.00	0.45	6.87	0.07	21.47	19.98	0.73
SAG-MED50	Ia.3/VIII	ERR599064 (TARA 093)	SAMEA2621779	PRJEB1787	SRF (5m)	South Pacific Ocean	32.76	0.33	6.00	0.06	24.55	16.38	0.74
AG-426-M19	Ib.1/III	ERR599030 (TARA 138)	SAMEA2623350	PRJEB1787	SRF (5m)	North Pacific Ocean	40.04	0.50	10.83	0.06	43.55	32.19	0.83
AG-426-M19	Ib.1/III	ERR599019 (TARA 048)	SAMEA2620404	PRJEB1787	SRF (5m)	Indian Ocean	37.78	0.52	9.44	0.06	62.90	22.33	0.77
AG-426-M19	Ib.1/III	ERR598969 (TARA 031)	SAMEA2619802	PRJEB1787	SRF (5m)	Red Sea	15.82	0.11	2.36	0.05	26.39	19.30	0.59
GOM-A1	Ib.1/III	ERR599030 (TARA 138)	SAMEA2623350	PRJEB1787	SRF (5m)	North Pacific Ocean	39.19	0.48	9.56	0.06	44.15	24.80	0.79
GOM-A1	Ib.1/III	ERR599019 (TARA 048)	SAMEA2620404	PRJEB1787	SRF (5m)	Indian Ocean	37.50	0.49	8.68	0.07	65.66	21.46	0.75
GOM-A1	Ib.1/III	ERR598969 (TARA 031)	SAMEA2619802	PRJEB1787	SRF (5m)	Red Sea	15.88	0.10	2.24	0.05	24.67	14.60	0.68
GOM-A5	Ib.1/III	ERR599030 (TARA 138)	SAMEA2623350	PRJEB1787	SRF (5m)	North Pacific Ocean	57.23	1.18	21.72	0.06	39.86	34.28	0.74
GOM-A5	Ib.1/III	ERR599019 (TARA 048)	SAMEA2620404	PRJEB1787	SRF (5m)	Indian Ocean	39.22	0.57	9.11	0.07	54.78	12.98	0.75
GOM-A5	Ib.1/III	ERR598969 (TARA 031)	SAMEA2619802	PRJEB1787	SRF (5m)	Red Sea	15.89	0.10	2.07	0.05	20.86	7.96	0.80
AG-325-E23	Ib.2/I	ERR598967 (TARA 096)	SAMEA2621859	PRJEB1787	SRF (5m)	South Pacific Ocean	46.85	0.91	14.58	0.08	21.92	18.83	0.78
AG-325-E23	Ib.2/I	ERR598993 (TARA 018)	SAMEA2619667	PRJEB1787	SRF (5m)	Mediterranean Sea	42.93	0.74	12.79	0.07	30.25	21.28	0.74
AG-325-E23	Ib.2/I	ERR598955 (TARA 004)	SAMEA2619376	PRJEB1787	SRF (5m)	North Atlantic Ocean	41.13	0.69	12.54	0.07	23.42	20.20	0.74
AG-359-O02	Ib.2/I	ERR598955 (TARA 004)	SAMEA2619376	PRJEB1787	SRF (5m)	North Atlantic Ocean	43.74	0.79	14.48	0.07	17.89	19.02	0.70
AG-359-O02	Ib.2/I	ERR598967 (TARA 096)	SAMEA2621859	PRJEB1787	SRF (5m)	South Pacific Ocean	49.54	0.88	14.40	0.08	24.93	25.01	0.77
AG-359-O02	Ib.2/I	ERR598993 (TARA 018)	SAMEA2619667	PRJEB1787	SRF (5m)	Mediterranean Sea	43.56	0.82	13.98	0.07	23.90	17.42	0.71
SAG-MED34	Ib.2/I	ERR598967 (TARA 096)	SAMEA2621859	PRJEB1787	SRF (5m)	South Pacific Ocean	50.00	1.14	17.70	0.08	19.84	17.33	0.73
SAG-MED34	Ib.2/I	ERR598993 (TARA 018)	SAMEA2619667	PRJEB1787	SRF (5m)	Mediterranean Sea	45.51	0.94	16.51	0.07	35.41	25.18	0.68
SAG-MED34	Ib.2/I	ERR598955 (TARA 004)	SAMEA2619376	PRJEB1787	SRF (5m)	North Atlantic Ocean	44.86	0.89	14.47	0.07	34.77	24.78	0.68
AAA288-G21	Ic.1	ERR599000 (TARA 076)	SAMEA2621232	PRJEB1787	MES (800m)	South Atlantic Ocean	45.65	1.52	9.61	0.17	12.51	15.42	0.79
AAA288-G21	Ic.1	ERR598964 (TARA 149)	SAMEA2623794	PRJEB1787	MES (740m)	North Atlantic Ocean	47.03	1.43	8.99	0.16	12.31	19.85	0.81
AAA288-G21	Ic.1	ERR599021 (TARA 064)	SAMEA2620815	PRJEB1787	MES (1000m)	Indian Ocean	23.76	0.41	2.57	0.17	15.14	16.73	0.78
AAA288-N07	Ic.1	ERR598964 (TARA 149)	SAMEA2623794	PRJEB1787	MES (740m)	North Atlantic Ocean	29.05	0.73	5.17	0.14	10.93	13.22	0.76
AAA288-N07	Ic.1	ERR599000 (TARA 076)	SAMEA2621232	PRJEB1787	MES (800m)	South Atlantic Ocean	26.82	0.68	4.80	0.15	9.26	8.58	0.51
AAA288-N07	Ic.1	ERR599021 (TARA 064)	SAMEA2620815	PRJEB1787	MES (1000m)	Indian Ocean	16.57	0.23	1.98	0.13	12.18	8.78	0.78
AG-464-P07	Ic.1	ERR599000 (TARA 076)	SAMEA2621232	PRJEB1787	MES (800m)	South Atlantic Ocean	39.55	1.14	7.55	0.16	9.17	15.87	0.72
AG-464-P07	Ic.1	ERR598964 (TARA 149)	SAMEA2623794	PRJEB1787	MES (740m)	North Atlantic Ocean	39.92	1.02	6.64	0.16	11.72	14.01	0.75
AG-464-P07	Ic.1	ERR599021 (TARA 064)	SAMEA2620815	PRJEB1787	MES (1000m)	Indian Ocean	22.95	0.38	2.48	0.15	13.08	9.76	0.77
AAA028-C07	IIIb	SRR3184739 (IR90)	SAMN04506996	PRJNA312830	SRF	Lake Klamath (California, USA)	22.74	0.91	2.44	0.16	149.87	10.79	0.60
AAA028-C07	IIIb	SRR948334 (LL 1011A)	SAMN02302397	PRJNA214105	SRF	Lake Lanier (Georgia, USA)	19.86	0.41	1.06	0.19	54.17	12.69	0.41
AAA028-C07	IIIb	SRR3184724 (Dex143)	SAMN04510817	PRJNA312985	SRF	Lake Dexter (Oregon, USA)	6.19	0.13	0.30	0.14	36.92	13.14	0.38

[1]SRF: surface; DCM: deep chlorophyll maximum; MES: mesopelagic.

[2]Percentage of polymorphic sites per gene

[3]Reads per Kilobase of Genome and Gigabase of Metagenome
