# Supplementary material for: The Evolutionary Success of the Marine Bacterium SAR11 Analyzed through a Metagenomic Perspective
Source: mSystems. 2020 Oct 6;5(5):e00605-20. doi: 10.1128/mSystems.00605-20 (PMC7542561; doi:10.1128/mSystems.00605-20)
Supplement: TABLE S3 [file mSystems.00605-20-st003.pdf]

Genome	Metagenome[1]	Depth	Isolation source	Polymorphic sites[2] (%)	pN	pS	pN/pS ratio	Abundance (RPKG[3])	γ/μ	Recombination coverage
Alteromonas macleodii AD45	SRR5788341 (Geotraces GA03)	MES	North Atlantic Ocean	13.72	0.25	0.57	0.29	56.21	4.15	0.43
Alteromonas macleodii AD45	SRR5788169 (Geotraces GA03)	SRF	North Atlantic Ocean	8.59	0.24	0.53	0.30	63.63	4.09	0.44
Alteromonas macleodii AD45	SRR5788344 (Geotraces GA03)	SRF	North Atlantic Ocean	3.09	0.08	0.20	0.22	44.18	3.47	0.44
Erythrobacter citreus LAMA 915	SAMN10839296	DCM	Mediterranean Sea	4.14	0.23	0.45	0.30	14.23	4.51	0.35
Erythrobacter citreus LAMA 915	ERR599112 (TARA_056)	MES	Indian Ocean	7.27	0.14	0.26	0.39	9.02	4.54	0.39
Erythrobacter citreus LAMA 915	ERR598999 (TARA_122)	MES	South Pacific Ocean	1.61	0.02	0.04	0.19	12.23	5.30	0.39
Ca . Nitrosopelagicus brevis CN25	SRR5788020 (Geotraces GA02)	MES	South Atlantic Ocean	33.63	1.65	2.90	0.78	172.24	17.12	0.58
Ca . Nitrosopelagicus brevis CN25	SRR5787994 (Geotraces GA03)	MES	North Atlantic Ocean	34.70	1.26	1.78	0.82	139.46	17.17	0.58
Ca . Nitrosopelagicus brevis CN25	SRR5788063 (Geotraces GA03)	MES	North Atlantic Ocean	21.37	0.48	0.73	0.70	73.04	13.26	0.53
MG-II Thalassoarchaea	SRR5788230 (Geotraces GA02)	MES	North Atlantic Ocean	22.36	0.47	0.49	0.61	41.22	17.03	0.34
MG-II Thalassoarchaea	SRR5788131 (Geotraces GA10)	SRF	South Atlantic Ocean	8.92	0.13	0.26	0.19	29.84	28.12	0.50
MG-II Thalassoarchaea	SRR5788284 (Geotraces GA02)	DCM	North Atlantic Ocean	5.71	0.09	0.17	0.36	37.28	15.22	0.33
Prochlorococcus marinus MED4	SRR5788024 (Geotraces GA02)	DCM	South Atlantic Ocean	47.97	1.83	2.42	0.82	203.15	48.80	0.79
Prochlorococcus marinus MED4	SRR5787990 (Geotraces GA02)	DCM	South Atlantic Ocean	42.79	1.48	2.03	0.79	195.22	61.11	0.80
Prochlorococcus marinus MED4	SRR5788058 (Geotraces GP13)	MES	South Pacific Ocean	44.62	1.47	1.99	0.71	144.10	56.86	0.81
Synechococcus sp. CC9902	SRR5788434 (Geotraces GA02)	SRF	North Atlantic Ocean	33.13	0.91	1.14	0.58	46.06	11.11	0.36
Synechococcus sp. CC9902	SRR5788234 (Geotraces GA02)	SRF	North Atlantic Ocean	12.80	0.26	0.36	0.53	25.10	8.92	0.39
Synechococcus sp. CC9902	SRR5788109 (Geotraces GA02)	SRF	North Atlantic Ocean	5.26	0.08	0.14	0.31	18.58	6.52	0.36

[1]SRF: surface; DCM: deep chlorophyll maximum; MES: mesopelagic.

[2]Percentage of polymorphic sites per gene.

[3]Reads per Kilobase of Genome and Gigabase of Metagenome.
