# Supplementary material for: The Evolutionary Success of the Marine Bacterium SAR11 Analyzed through a Metagenomic Perspective
Source: mSystems. 2020 Oct 6;5(5):e00605-20. doi: 10.1128/mSystems.00605-20 (PMC7542561; doi:10.1128/mSystems.00605-20)
Supplement: FIG S2 [file mSystems.00605-20-sf002.pdf]

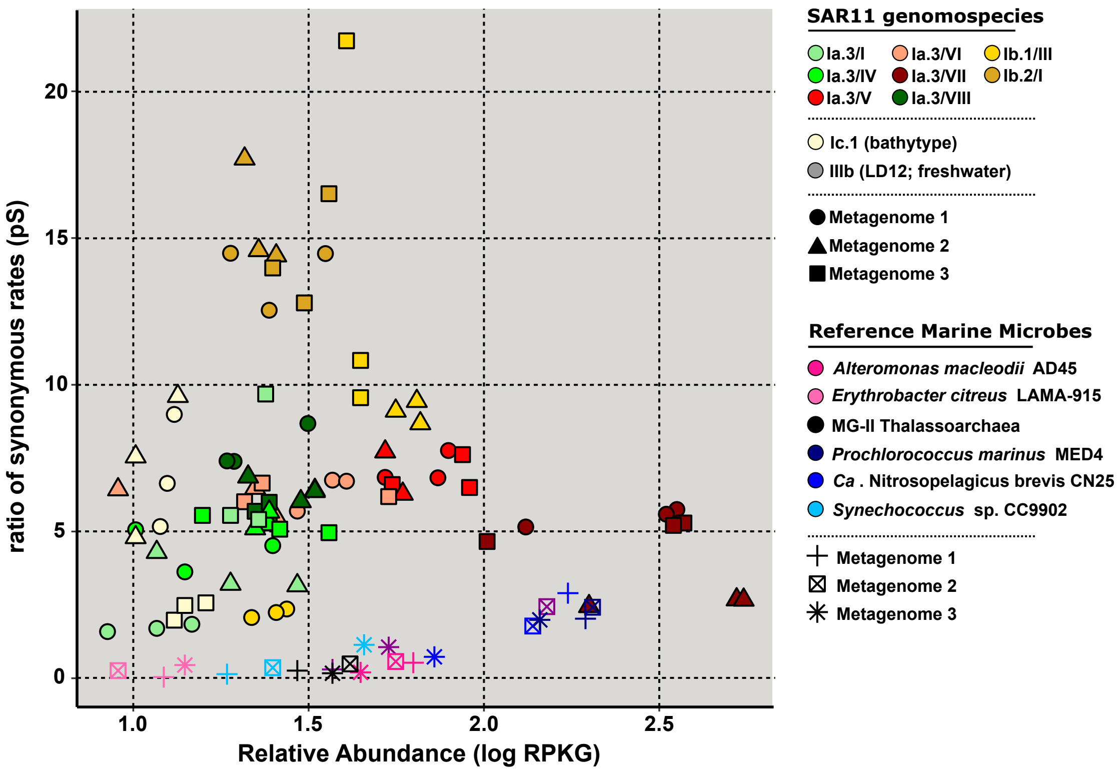

ratio of synonymous rates (pS)
0
5
10
15
20
1.0
1.5
2.0
2.5
Relative Abundance (log RPKG)
SAR11 genomospecies
Ia.3/I
Ia.3/VI
Ib.1/III
Ia.3/IV
Ia.3/VII
Ib.2/I
Ia.3/V
Ia.3/VIII
Ic.1 (bathytype)
IIIb (LD12; freshwater)
Metagenome 1
Metagenome 2
Metagenome 3
Reference Marine Microbes
Alteromonas macleodii AD45
Erythrobacter citreus LAMA-915
MG-II Thalassoarchaea
Prochlorococcus marinus MED4
Ca . Nitrosopelagicus brevis CN25
Synechococcus sp. CC9902
Metagenome 1
Metagenome 2
Metagenome 3
