# Supplementary material for: The Evolutionary Success of the Marine Bacterium SAR11 Analyzed through a Metagenomic Perspective
Source: mSystems. 2020 Oct 6;5(5):e00605-20. doi: 10.1128/mSystems.00605-20 (PMC7542561; doi:10.1128/mSystems.00605-20)
Supplement: TABLE S4 [file mSystems.00605-20-st004.pdf]

Group	Genomospecies	Genome	Genome Size (bp)	Completeness (%)	Contamination (%)	Isolation source	Depth	Type	Accession number	Biosample	Bioproject
SAR11	Ia.3/I	AG-325-C10	1,087,529	78.29	0	South Pacific Ocean	14m	SAG	GCA_902515085.1	SAMEA6077249	PRJEB33281
SAR11	Ia.3/I	HTCC7211	1,456,888	100	0.47	Sargasso Sea, BATS	1m	Isolate	GCA_000155895.1	SAMN02436224	PRJNA19339
SAR11	Ia.3/I	HTCC7214	1,375,060	99.05	0.47	Sargasso Sea, BATS	1m	Isolate	GCA_000701385.1	SAMN02841172	PRJNA219140
SAR11	Ia.3/IV	HTCC8051	1,395,018	99.76	0	Oregon Coast	5m	Isolate	GCA_000472605.1	SAMN02440710	PRJNA182738
SAR11	Ia.3/IV	HTCC9022	1,360,964	100	0	--	--	Isolate	GCA_000472565.1	SAMN02440781	PRJNA182740
SAR11	Ia.3/IV	SAG-MED49	1,167,552	85.47	0.95	Mediterranean Sea	5m	SAG	--	SAMN15685207	PRJNA473343
SAR11	Ia.3/V	AAA795-A20	1,140,609	86.82	0	Red Sea	1m	SAG	GCA_001179845.1	SAMEA3368553	PRJEB9287
SAR11	Ia.3/V	AG-422-D23	1,279,565	90.28	0	North Atlantic Ocean	57.7m	SAG	GCA_902571845.1	SAMEA6076235	PRJEB33281
SAR11	Ia.3/V	HIMB083	1,395,997	99.53	0	--	--	Isolate	GCA_000504225.1	SAMN02597166	PRJNA74601
SAR11	Ia.3/VI	AAA797-I19	1,016,895	67.04	0.47	Red Sea	1m	SAG	GCA_001180225.1	SAMEA3368572	PRJEB9287
SAR11	Ia.3/VI	AG-390-A02	1,079,061	73.58	0	North Atlantic Ocean	8m	SAG	GCA_902554465.1	SAMEA6075582	PRJEB33281
SAR11	Ia.3/VI	AG-390-M19	873,216	59.18	0	North Atlantic Ocean	8m	SAG	GCA_902558955.1	SAMEA6075806	PRJEB33281
SAR11	Ia.3/VII	AG-414-O11	1,212,678	90.05	0	North Atlantic Ocean	119m	SAG	GCA_902570235.1	SAMEA6076036	PRJEB33281
SAR11	Ia.3/VII	AG-430-E20	1,183,991	90.6	0	North Atlantic Ocean	89.9m	SAG	GCA_902580005.1	SAMEA6079829	PRJEB33281
SAR11	Ia.3/VII	SAG-MED22	1,191,160	98.56	0.52	Mediterranean Sea	5m	SAG	--	SAMN15685184	PRJNA473343
SAR11	Ia.3/VIII	AG-390-D18	1,218,852	86.05	0	North Atlantic Ocean	8m	SAG	GCA_902555825.1	SAMEA6075651	PRJEB33281
SAR11	Ia.3/VIII	AG-390-L03	1,352,095	94.31	0	North Atlantic Ocean	8m	SAG	GCA_902558255.1	SAMEA6075772	PRJEB33281
SAR11	Ia.3/VIII	SAG-MED50	1,257,069	89.65	0.24	Mediterranean Sea	5m	SAG	--	SAMN15685208	PRJNA473343
SAR11	Ib.1/III	AG-426-M19	1,256,227	84.59	0	North Atlantic Ocean	90.8m	SAG	GCA_902578885.1	SAMEA6079697	PRJEB33281
SAR11	Ib.1/III	GOM-A1	1,330,051	93.98	1.2	Gulf of Mexico	1m	SAG	GCA_001704065.1	SAMN03944292	PRJNA291283
SAR11	Ib.1/III	GOM-A5	655,485	52.42	0.07	Gulf of Mexico	1m	SAG	GCA_001704055.1	SAMN03944296	PRJNA291283
SAR11	Ib.2/I	AG-325-E23	1,159,533	67.92	0	South Pacific Ocean	14m	SAG	GCA_902515595.1	SAMEA6077300	PRJEB33281
SAR11	Ib.2/I	AG-359-O02	1,098,312	89.63	0	North Atlantic Ocean	10m	SAG	GCA_902543155.1	SAMEA6069045	PRJEB33281
SAR11	Ib.2/I	SAG-MED34	796,940	62.7	0	Mediterranean Sea	5m	SAG	--	SAMN15685195	PRJNA473343
SAR11	Ic.1	AAA288-G21	909,786	55.62	0.11	North Pacific Ocean	770m	SAG	GCA_000513075.1	SAMN02597281	PRJNA232476
SAR11	Ic.1	AAA288-N07	954,664	71.93	1.2	North Pacific Ocean	770m	SAG	GCA_000513055.1	SAMN02597280	PRJNA232477
SAR11	Ic.1	AG-464-P07	1,138,562	77.71	0	South Pacific Ocean	203m	SAG	GCA_902608355.1	SAMEA6079275	PRJEB33281
SAR11	IIIb	AAA028-C07	846,566	74.12	0.11	Lake Mendota	--	SAG	GCA_000419465.1	SAMN02441251	PRJNA76829
Reference Marine Microbes	--	*A. macleodii* AD45	4,674,885	100	0	Mediterranean Sea	5m	Isolate	GCA_000300175.1	SAMN02604120	PRJNA65405
Reference Marine Microbes	--	*E. citreus* LAMA-915	3,094,736	100	0	South Altlantic Ocean	3600m	Isolate	GCA_001235865.1	SAMN03344099	PRJNA193448
Reference Marine Microbes	--	*Synechococcus* sp. CC9902	2,234,828	100	0	Pacific Ocean	5m	Isolate	GCA_000012505.1	SAMN02598312	PRJNA13655
Reference Marine Microbes	--	*P. marinus* MED4	1,657,990	100	0	Mediterranean Sea	5m	Isolate	GCA_000011465.1	SAMEA3138209	PRJNA213
Reference Marine Microbes	--	*Ca*. Nitrosopelagicus brevis CN25	1,232,128	100	0	North Pacific Ocean	25m	Isolate	GCA_000812185.1	SAMN03273964	PRJNA223412
Reference Marine Microbes	--	MG-II Thalassoarchaeota	2,870,682	88.3	2.1	Mediterranean Sea	50m	MAG	--	SAMN02954236	PRJNA257723
